# Artificial Intelligence for COVID-19: A Systematic Review

**DOI:** 10.3389/fmed.2021.704256

**Published:** 2021-09-30

**Authors:** Lian Wang, Yonggang Zhang, Dongguang Wang, Xiang Tong, Tao Liu, Shijie Zhang, Jizhen Huang, Li Zhang, Lingmin Chen, Hong Fan, Mike Clarke

**Affiliations:** ^1^Department of Respiratory and Critical Care Medicine, West China Hospital/West China School of Medicine, Sichuan University, Chengdu, China; ^2^Department of Periodical Press and National Clinical Research Center for Geriatrics, West China Hospital, Sichuan University, Chengdu, China; ^3^Chinese Evidence-Based Medicine Center, West China Hospital, Sichuan University, Chengdu, China; ^4^Department of Anesthesiology and National Clinical Research Center for Geriatrics, West China Hospital, Sichuan University and The Research Units of West China, Chinese Academy of Medical Sciences, Chengdu, China; ^5^Northern Ireland Methodology Hub, Queen's University Belfast, Belfast, United Kingdom

**Keywords:** artificial intelligence, COVID-19, diagnosis, prognosis evaluation, epidemic prediction, drug discovery 2

## Abstract

**Background:** Recently, Coronavirus Disease 2019 (COVID-19), caused by severe acute respiratory syndrome virus 2 (SARS-CoV-2), has affected more than 200 countries and lead to enormous losses. This study systematically reviews the application of Artificial Intelligence (AI) techniques in COVID-19, especially for diagnosis, estimation of epidemic trends, prognosis, and exploration of effective and safe drugs and vaccines; and discusses the potential limitations.

**Methods:** We report this systematic review following the Preferred Reporting Items for Systematic Reviews and Meta-Analyses (PRISMA) guidelines. We searched PubMed, Embase and the Cochrane Library from inception to 19 September 2020 for published studies of AI applications in COVID-19. We used PROBAST (prediction model risk of bias assessment tool) to assess the quality of literature related to the diagnosis and prognosis of COVID-19. We registered the protocol (PROSPERO CRD42020211555).

**Results:** We included 78 studies: 46 articles discussed AI-assisted diagnosis for COVID-19 with total accuracy of 70.00 to 99.92%, sensitivity of 73.00 to 100.00%, specificity of 25 to 100.00%, and area under the curve of 0.732 to 1.000. Fourteen articles evaluated prognosis based on clinical characteristics at hospital admission, such as clinical, laboratory and radiological characteristics, reaching accuracy of 74.4 to 95.20%, sensitivity of 72.8 to 98.00%, specificity of 55 to 96.87% and AUC of 0.66 to 0.997 in predicting critical COVID-19. Nine articles used AI models to predict the epidemic of the COVID-19, such as epidemic peak, infection rate, number of infected cases, transmission laws, and development trend. Eight articles used AI to explore potential effective drugs, primarily through drug repurposing and drug development. Finally, 1 article predicted vaccine targets that have the potential to develop COVID-19 vaccines.

**Conclusions:** In this review, we have shown that AI achieved high performance in diagnosis, prognosis evaluation, epidemic prediction and drug discovery for COVID-19. AI has the potential to enhance significantly existing medical and healthcare system efficiency during the COVID-19 pandemic.

## Introduction

Coronavirus Disease 2019 (COVID-19), caused by severe acute respiratory syndrome virus 2 (SARS-CoV-2) was first detected in December 2019, and spread rapidly to most cities and countries around the world (1–3). During face-to-face contact, SARS-CoV-2 is mainly transmitted through respiratory droplets (4). The infection may cause mild symptoms of upper respiratory tract infections, as well as extremely severe sepsis and shock. It may lead to serious and lethal complications in vulnerable populations, especially in the elderly with comorbidities (4–6). As of 16 March 2021, SARS-CoV-2 has affected more than 200 countries and led to enormous losses, causing more than 120 million confirmed cases and 2.6 million identified deaths. The rising incidence and massive casualties caused by COVID-19 exert considerable pressure on limited healthcare resources. Effective tools are needed to streamline the diagnosis, treatment and surveillance of COVID-19 and increase the clinical efficiency of healthcare systems (7). Recent studies have shown that artificial intelligence is a promising technology as they can achieve better scale-up, accelerate processing power, and even outperform humans in specific healthcare tasks (8).

Artificial intelligence (AI) is a field of algorithm-based applications that enable machines to solve knowledge problems and use algorithms to simulate human decision-making, and continuously improves performance by applying inputted data to perform specific tasks (9–11). The advantages of AI are reflected in high sensitivity and specificity in identifying the object, the speed of reporting and consistency of results (9). In recent years, AI has made significant progress, especially in predictive machine learning models for medical care. Deep learning is a method of ML, based on the complex architectures of Artificial Neural Networks (ANN). Deep learning reveals significant discriminative performance after providing sufficient training data sets and is essential for making predictions (12). In medicine, technologies based on Artificial intelligence and machine learning (AI/ML) aim to improve the quality of medical care, increase diagnostic accuracy and reduce potential errors and predict outcomes by discovering new insights from the enormous amount of data produced by the experience of many patients (10).

Researchers have made significant contributions to the campaign against COVID-19, and new COVID-19-related AI models in the literature are rapidly increasing. Well-trained artificial intelligence models can ensure accurate and rapid diagnosis or assist doctors to streamline the diagnosis and reduce manual labor (13, 14). AI models could early detect the patients at higher risk and characterize the epidemiology of COVID-19 and model disease transmission by training data (15, 16). Artificial intelligence-based methods could assist in the discovery of novel drugs and vaccines, such as repurpose exist drugs, screen targets as vaccines based on the potential mutation model to SARS-CoV-2, as well as screen compounds as potential adjuvants for vaccines (3, 17). AI-powered chatbots have been used with success in clinical scenarios and can advise many more people than a manned call center and ease the stress placed on medical hotlines (18). AI could manage the pandemic by using thermal imaging to scan public spaces for people potentially infected, and by enforcing social distancing and lockdown measures (3, 17).

Artificial intelligence has been widely used in COVID-19, including diagnosis, public health, clinical decision making, social control, therapeutics, vaccine development, surveillance, combination with big data, operation of other core clinical services, and management of patients with COVID-19 (3, 18, 19). In order to solve the significant pressure of the limited medical resources caused by the pandemic of COVID-19, rapid diagnosis, accurate prediction, enhanced monitoring, and effective treatments are the most important measures to control the spread of the pandemic. Many related review articles have been published. However, the results of these studies are inconsistent and there is little research systematically assessing the application of AI for COVID-19 in accordance with PRISMA, and most of them only discuss aspects such as diagnosis or treatment. Therefore, we conducted this review to assess the performance of AI for COVID-19 systematically, and to describe the main categories of AI use, the potential benefits and limitations and future directions for AI.

## Methods

We registered the protocol for this review in advance (PROSPERO CRD42020211555, URL: https://www.crd.york.ac.uk/prospero/).

### Search Strategy and Eligibility Criteria

This systematic review is reported in accordance with the Preferred Reporting Items for Systematic Reviews and Meta-Analyses (PRISMA) guidelines (20) (Supplementary Material 2). We searched PubMed, Embase and the Cochrane Library for published studies from the inception of these resources to 19 September 2020 using the following terms related to artificial intelligence and COVID-19: “Artificial intelligence,” “Machine Intelligence,” “Machine learning,” “Deep learning,” “Predictive model,” “2019 novel coronavirus disease,” “COVID-19,” “2019 novel coronavirus infection,” “coronavirus disease-19,” and “2019-nCoV disease.” The details of the search strategy are in the Supplementary Material 1.

We included original studies fulfilling the following criteria: (I) research topic was focused on the application of AI for COVID-19, (II) participants had a confirmed diagnosis of COVID-19 by reverse transcription-polymerase chain reaction (RT-PCR) testing or other laboratory examination (where appropriate), and (III) article was published in English.

We excluded studies if: (I) insufficient data were available, (II) we were unable to access the full text or complete data, or (III) the report was a review, case-report or comment.

Two trained researchers (Lian Wang, Dongguang Wang) screened titles, abstracts and the full text of potentially eligible studies independently using Endnote X8.2 software, Thomson Reuters. Discrepancies were resolved through consultation with a third researcher (Xiang Tong, Tao Liu).

### Data Abstraction and Quality Assessment

We extracted data and recorded the following information for each study: basic information for the article (title, first author, date of publication), experimental design (algorithm, sample size) and primary outcome (sensitivity, accuracy and specificity of AI for diagnosis and prognosis evaluation; prediction of epidemic; drug repurposing and development). If a study used multiple models, we extracted the most discriminative one.

Three researchers (SZ, JH, LZ) used PROBAST (prediction model risk of bias assessment tool) to assess the risk of bias in the included studies (21). The PROBAST statement was divided into four domains: participants, predictors, outcome, and analysis. These domains contain a total of 20 signal questions to help structure judgment of risk of bias for prediction models, such as the range of included patients, whether the same predictors and results were defined for all participants, whether the clinical decision rule was determined prospectively, and whether a relevant measure of accuracy was reported (22). The details of PROBAST are in the Supplementary Material 3.

We divided the included studies into four categories: diagnosis, prognosis, epidemic prediction, and drug discovery. In the diagnosis and prognosis domains, we evaluated the classification performance using AUC, accuracy, sensitivity, and specificity. For epidemic trends and drugs and vaccines discovery, we listed the results only because there are no suitable evaluation indicators.

### Role of the Funding Source

This study was supported by National Key R&D Program of China (2017YFC1309703) and 1·3·5 project for disciplines of excellence–Clinical Research Incubation Project, West China Hospital, Sichuan University (2019HXFH008). The funders of this research did not contribute to the study design, data analysis, data interpretation or preparation of the manuscript.

## Results

**Figure 1:**
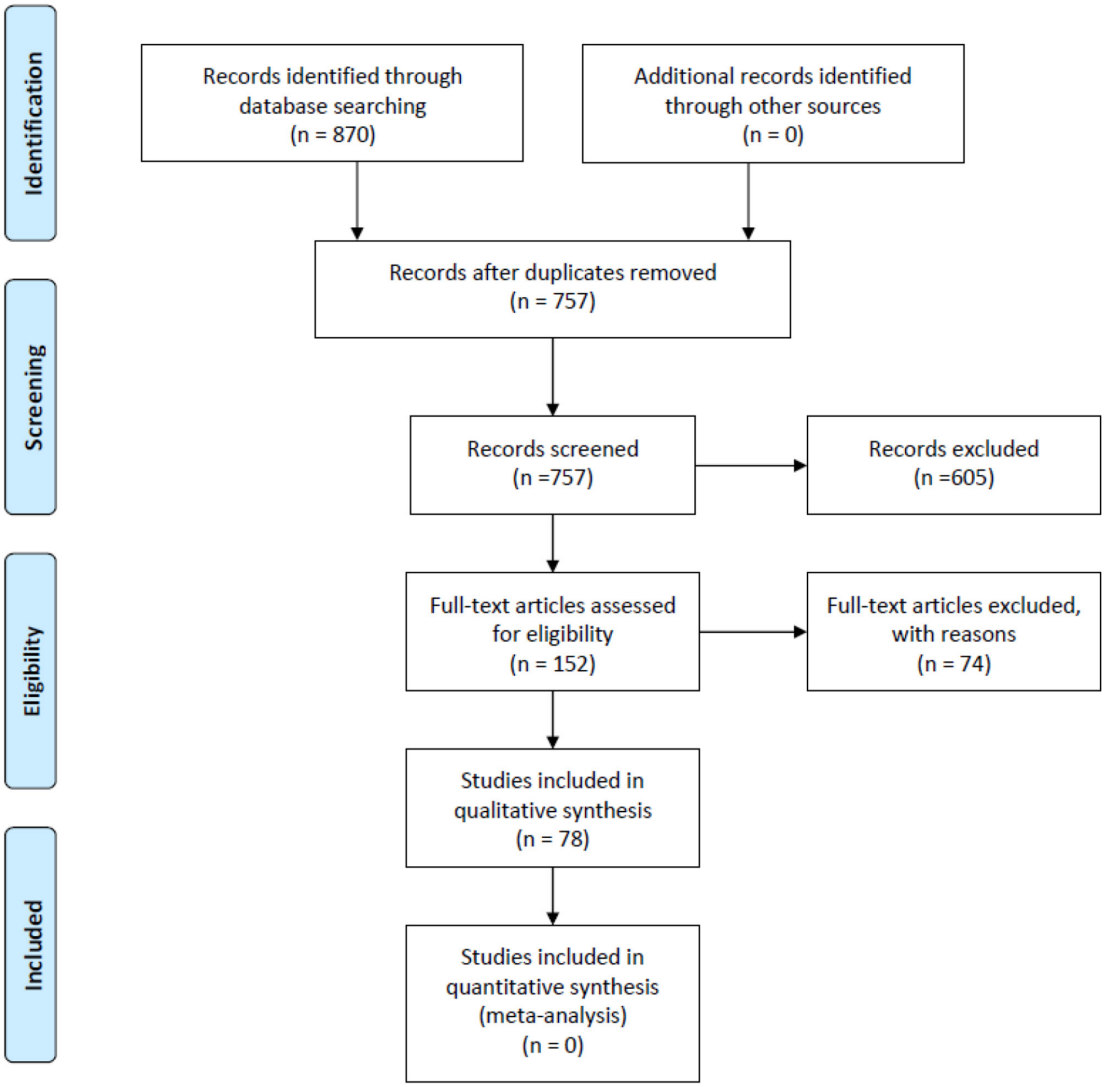
PRISMA flow diagram for the literature search and study selection.

We retrieved a total of 870 records from PubMed, Embase and the Cochrane Library. Of these, 78 studies met our inclusion criteria. Details of the study selection process are shown in Figure 1. Among the 78 included studies (12, 14, 17, 23–97), 46 discussed AI-assisted diagnosis of COVID-19 (14, 23–67), 14 evaluated prognosis (12, 68–80), 9 estimated infected cases, infection rates and epidemic trends (81–89), 8 explored potential effective and safe drugs (90–97), primarily through drug repurposing and drug development and 1 article predicted vaccine targets that has the potential to develop COVID-19 vaccines (17).

We used PROBAST to evaluate the quality of the 60 articles related to diagnosis or prognosis for COVID-19 (Table 1) (12, 14, 23–80). According to the assessment with PROBAST, all models had a high risk of bias. In the absence of appropriate evaluation tools for the other 18 articles, the quality of these was not assessed (17, 81–97).

**Table 1:** Risk of bias assessment (by PROBAST).

	**Risk of bias**			
**References**	**Participants**	**Predictors**	**Outcome**	**Analysis**
**Diagnosis**				
Abbasian Ardakani et al. (23)	High	Unclear	Low	High
Bai et al. (14)	High	Unclear	Low	High
Ardakani et al. (24)	High	Unclear	Low	High
Han et al. (25)	High	Unclear	Low	High
Ko et al. (26)	High	High	Low	High
Li et al. (27)	High	Unclear	Low	High
Liu et al. (28)	High	Unclear	Low	High
Mei et al. (29)	High	Unclear	Unclear	High
Mishra et al. (30)	High	High	High	High
Ouyang et al. (31)	High	Unclear	Low	High
Sakagianni et al. (32)	High	High	High	High
Sharma (33)	High	High	High	High
Wang et al. (34)	High	High	High	High
Wang et al. (35)	Low	High	Low	High
Wu et al. (36)	High	Unclear	High	High
Yan et al. (37)	High	Unclear	Low	High
Zhang et al. (38)	Low	High	High	High
Harmon et al. (39)	Low	Unclear	High	High
Jaiswal et al. (40)	High	High	High	High
Ni et al. (41)	Low	Unclear	High	High
Song et al. (42)	Low	High	Low	High
Xu et al. (43)	Low	High	Low	High
Yang et al. (44)	Low	Low	Low	High
Apostolopoulos and Mpesiana (45)	High	High	High	High
Das et al. (47)	High	High	High	High
Khan et al. (49)	High	High	High	High
Mahmud et al. (50)	High	High	High	High
Murphy et al. (51)	High	Unclear	Low	High
Ouchicha et al. (52)	High	High	High	High
Ozturk et al. (53)	High	High	High	High
Togaçar et al. (54)	High	High	High	High
Vaid et al. (55)	High	High	High	High
Elaziz et al. (48)	Low	Unclear	Low	High
Bressem et al. (46)	High	High	High	High
Altan and Karasu (56)	High	High	High	High
Brunese et al. (57)	High	High	High	High
Che Azemin et al. (58)	High	High	High	High
Islam et al. (59)	High	High	High	High
Jain et al. (60)	High	High	High	High
Nour et al. (61)	High	High	High	High
Rahaman et al. (62)	High	High	High	High
Rahimzadeh and Attar (63)	High	High	High	High
Rajaraman et al. (64)	High	High	High	High
Toraman et al. (65)	High	High	High	High
Ucar and Korkamz (66)	High	High	High	High
Waheed et al. (67)	High	High	High	High
**Prognosis**				
Assaf et al. (68)	High	High	High	High
Li et al. (70)	High	Unclear	Low	High
Liang et al. (71)	High	High	Low	High
Yao et al. (75)	High	High	High	High
Yu et al. (76)	High	High	High	High
Iwendi et al. (69)	High	High	High	High
Abdulaal et al. (12)	High	High	Low	High
Ma et al. (72)	High	High	Low	High
Mushtaq et al. (73)	High	High	Low	High
Wu et al. (77)	High	High	High	High
Cheng et al. (78)	High	Unclear	Low	High
Fu et al. (79)	High	High	High	High
Wu et al. (74)	High	Low	Low	High
Xiao et al. (80)	High	Low	Low	High

*PRISMA, the Preferred Reporting Items for Systematic Reviews and Meta-Analyses; PROBAST, prediction model risk of bias assessment tool*.

### AI-Assisted Diagnosis for COVID-19

We included 46 studies related to AI-assisted diagnosis through chest images for COVID-19 (14, 23–67). The findings of these studies ranged as follows, total accuracy: 70.00 to 99.92%, sensitivity: 73.00 to 100.00%, specificity: 25 to 100.00%, AUC: 0.732 to 1.000.

### Chest CT Images

Deep learning with a convolutional neural network (CNN) has gained increasing attention for its outstanding image recognition performance (98). Several of the studies (*n* = 18) we identified had developed AI models based on CNN and these showed excellent ability to discriminate COVID-19 and non-COVID pneumonia by automatically detecting chest CT images with an accuracy of 70.00 to 99.87%, sensitivity of 73.00 to 100.00%, specificity of 25 to 100.00%, and AUC of 0.732 to 1.000 (Table 2) (14, 23, 24, 26, 27, 29–31, 33–40, 43, 44). Mei et al. (29) developed a joint CNN model that diagnoses COVID-19 patients rapidly by combining chest CT findings with clinical symptoms, exposure history, and laboratory tests. Moreover, Mishra et al. (30) proposed a decision-fusion approach, which combined the predictions of each Deep CNN model and achieved results above 86% for all the performance metrics under consideration. Three studies (14, 41, 42) found that AI models had higher test accuracy, sensitivity, specificity than radiologists, and with the assistance of AI, the radiologists made diagnosis with much faster speeds and achieved a higher diagnostic performance.

**Table 2:** Application of AI in using chest CT images to diagnose COVID-19.

**References**	**Algorithm**	**Subjects**	**Objective**	**Results**
Abbasian Ardakani et al. (23)	CAD	612 patients (306 COVID-19)	Identify COVID-19/non-COVID-19	Accuracy, 91.94%; Sensitivity, 93.54%; Specificity, 90.32%; AUC, 0.965
Bai et al. (14)	EfficientNet B4	1,186 patients (521 COVID-19)	Identify COVID-19/non-COVID-19	Accuracy, 96%; Sensitivity, 95%; Specificity, 96%; AUC, 0.95
Ardakani et al. (24)	ResNet-101	194 patients (108 COVID-19)	Identify COVID-19/non-COVID-19	Accuracy, 99.51%; Sensitivity, 100%; Specificity, 99.02%; AUC, 0.994
Han et al. (25)	AD3D-MIL	460 chest CT images (230 COVID-19)	Identify COVID-19/non-COVID-19	Accuracy, 97.9%; Sensitivity, 97.9%; AUC, 0.99; Precision, 97.9%; F1 score 97.9%
Ko et al. (26)	FCONet	2,551 chest CT images (1,194 COVID-19)	Identify COVID-19/other pneumonia/non-pneumonia	Accuracy, 99.87%; Sensitivity, 99.58%; Specificity, 100%; AUC, 1.00
Li et al. (27)	COVNet	4,356 chest CT images (1,296 COVID-19)	Identify COVID-19/CAP/non-pneumonia	Sensitivity, 90%; Specificity, 96%; AUC, 0.96
Liu et al. (28)	EBT	100 patients (73 COVID-19)	Identify COVID-19/general pneumonia	Accuracy, 94.16%; Sensitivity, 88.62%; Specificity, 100%; AUC, 0.99
Mei et al. (29)	CNN	905 patients (419 COVID-19)	Identify COVID-19/non-COVID-19	Accuracy, 79.6%; Sensitivity, 83.6%; Specificity, 75.9%; AUC, 0.86
Mishra et al. (30)	DCNN	727 chest CT images (360 COVID-19)	Identify COVID-19/non-COVID-19	Accuracy, 88.34%; Sensitivity, 88.13%; Specificity, 90.51%; AUC, 0.883; F1 score 86.7%
Ouyang et al. (31)	Attention RN34 + DS (3D CNN)	4,982 chest CT images (3,389 COVID-19)	Identify COVID-19/CAP	Accuracy, 87.5%; Sensitivity, 86.9%; Specificity, 90. 1%; AUC, 0.944; F1 score 82%
Sakagianni et al. (32)	AutoML Cloud Vision	746 chest CT images (349 COVID-19)	Identify COVID-19/non-COVID-19	Sensitivity, 88.31%; Precision, 88.31%
Sharma (33)	ResNet	2,200 chest CT images (800 COVID-19)	Identify COVID-19/viral pneumonia	Accuracy, 91%; Sensitivity, 92.1%; Specificity, 90.29%
Wang et al. (34)	3D-ResNet (DCNN)	4,657 chest CT images (1,315 COVID-19)	Identify COVID-19/viral pneumonia	Accuracy, 93.3 ± 0.8%; Sensitivity, 87.6 ± 4.3%; Specificity, 95.5 ± 2.1%; AUC, 97.3 ± 1.1; Precision, 88.4 ± 4.1%; F1 score 87.8 ± 1.5%
Wang et al. (35)	DenseNet121-FPN/COVID-19Net	1,266 chest CT images (924 COVID-19)	Identify COVID-19/viral pneumonia /other pneumonia	Accuracy, 80.12%; Sensitivity, 79.35%; Specificity, 81.16%; AUC, 0.88; F1 score 82.02%
Wu et al. (36)	ResNet50	495 patients (368 COVID-19)	Identify COVID-19 /other pneumonia	Accuracy, 70%; Sensitivity, 73%; Specificity, 61.5%; AUC, 0.732
Yan et al. (37)	MSCNN	828 chest CT images (416 COVID-19)	Identify COVID-19/common pneumonia	Accuracy, 97.7%; Sensitivity, 99.5%; Specificity, 95.6%; AUC, 0.962
Zhang et al. (38)	AI-based system	260 patients (83 COVID-19)	Identify COVID-19/common pneumonia/normal pneumonia/normal	Accuracy, 92.49%; Sensitivity, 94.93%; Specificity, 91.13%; AUC, 0.9813
Harmon et al. (39)	3D models (based on Densnet-121)	2,617 patients (922 COVID-19)	Identify COVID-19/non-COVID-19	Accuracy, 90.8%; Sensitivity, 84%; Specificity, 93%; AUC, 0.949
Jaiswal et al. (40)	DTL with DenseNet201	2,492 CT scan images (1,262 COVID-19)	Identify COVID-19/non-COVID-19	Accuracy, 96.25%; Sensitivity, 96.29%; Specificity, 96.21%; AUC, 0.97
Ni et al. (41)	Convolutional MVP-Net, 3D U-Net	14,435 patients (2,154 COVID-19)	Identify COVID-19/non-COVID-19	For patient level, Accuracy, 94%; Sensitivity, 100%; Specificity, 25%; AUC, 0.86. For lobe level, Accuracy, 82%; Sensitivity, 96%; Specificity, 63%; AUC, 0.87
Song et al. (42)	BigBiGAN	201 patients (98 COVID-19)	Identify COVID-19/non-COVID-19	Sensitivity, 92%; Specificity, 91%
Xu et al. (43)	CNN	509 patients (110 COVID-19)	Identify COVID-19/ IAVP/non-COVID-19	Accuracy, 86.7%; Sensitivity, 86.7%; Specificity, 81.2%; F1 score 83.9%
Yang et al. (44)	DenseNet	295 patients (146 COVID-19)	Identify COVID-19/normal	Accuracy, 92%; Sensitivity, 97%; Specificity, 87%; AUC, 0.98; F1 score 93%

*CNN, Convolutional Neural Network; DFCNN, Dense Fully Convolutional Neural Network; EBT, Ensemble of Bagged Tree; DCNN, Dense Convolutional Neural Network; MSCNN, Multi-Scale Convolutional Neural Network; DTL, Deep Transfer Learning; IAVP, Influenza-A Viral Pneumonia*.

In addition, some studies reported applications distinguishing COVID-19 from non-COVID-19 or other pneumonia by using the AD3D-MIL model (25), Ensemble of Bagged Tree (28), AutoML Cloud Vision (32). All of these performed well.

### Chest X-Ray Images

Although CT images have high sensitivity in detecting COVID-19, costs and radiation doses are relatively high. On the contrary, chest X-ray is a low-cost and rapid detection method, which might be used in initial screening of suspected cases of COVID-19 infection, supporting the timely application of quarantine measures in positive patients (45). Several studies have developed AI techniques to automatically detect and extract features from chest X-rays to assist in the diagnosis of COVID-19 with high accuracy (71.90 to 99.92%), sensitivity (75.00 to 99.44%), specificity (71.80 to 100.00%), and AUC (0.81 to 0.999) (45–67) (Table 3).

**Table 3:** Application of AI in using chest x-ray images to diagnose COVID-19.

**References**	**Algorithm**	**Subjects**	**Objective**	**Results**
Apostolopoulos and Mpesiana (45)	MobileNet v2	1,442 chest x-ray images (224 COVID-19)	Identify COVID-19/viral/bacterial/ pneumonia/normal	Accuracy, 96.78%; Sensitivity, 98.66%; Specificity, 96.46%
Das et al. (47)	Truncated Inception Net	6,845 chest x-ray images (162 COVID-19)	Identify COVID-19/non-COVID-19	Accuracy, 99.92%; Sensitivity, 93%; Specificity, 100%; AUC, 0.99; Precision, 100%; F1 score 96%
Khan et al. (49)	CoroNet	3,084 chest x-ray images (290 COVID-19)	Identify COVID-19/viral/bacterial/ pneumonia/normal	Accuracy, 89.60%; Sensitivity, 89.92%; Specificity, 96.4%; Precision, 90%; F1 score 89.8%
Mahmud et al. (50)	CovXNet	6,161 chest x-ray images (305 COVID-19)	Identify COVID-19/viral/bacterial/ pneumonia/normal	Accuracy, 90.2%; Sensitivity, 89.9%; Specificity, 89.1%; AUC, 0.911; Precision, 90.8%; F1 score 90.4%
Murphy et al. (51)	CAD4COVID-XRay	24,678 chest x-ray images (730 COVID-19)	Identify COVID-19/non-COVID-19	Sensitivity, 75%; Specificity, 78%; AUC, 0.81
Ouchicha et al. (52)	CVDNet	2,905 chest x-ray images (219 COVID-19)	Identify COVID-19/viral pneumonia/normal	Accuracy, 96.69%; Sensitivity, 96.84%; Precision 96.72%; F1 score 96.68%
Ozturk et al. (53)	DarkCovidNet	1,127 chest x-ray images (127 COVID-19)	Identify COVID-19/pneumonia/normal	Accuracy, 98.08%; Sensitivity, 95.13%; Specificity, 95.30%; Precision, 98.03%; F1 score 96.51%
Togaçar et al. (54)	MobileNetV2, SqueezeNet	458 chest x-ray images (295 COVID-19)	Identify COVID-19/pneumonia/normal	Accuracy, 99.34%; Sensitivity, 99.32%; Specificity, 99.37%; AUC, 0.982; Precision, 99.66%; F1 score 99.49%
Vaid et al. (55)	CNN	545 chest x-ray images (181 COVID-19)	Identify COVID-19/ normal	Accuracy>96.3%; Sensitivity, 97.1%; Precision 91.7%; F1 score 94.3%
Elaziz et al. (48)	FrMEMs	1,891 chest x-ray images (216 COVID-19)	Identify COVID-19/non-COVID-19	Accuracy, 96.09%; Sensitivity, 98.75%; Precision 98.75%
Bressem et al. (46)	DenseNet-121	46,754 chest x-ray images (196 COVID-19)	Identify COVID-19/non-COVID-19/normal	Sensitivity, 93%; Specificity, 100%
Altan and Karasu (56)	EfficientNet-B0	2,905 chest x-ray images (219 COVID-19)	Identify COVID-19/viral pneumonia/normal	Accuracy, 99.69%; Sensitivity, 99.44%; Specificity, 99.81%; Precision, 99.62%; F1 score 99.53%
Brunese et al. (57)	VGG16	6,523 chest x-ray images (250 COVID-19)	Identify COVID-19/pulmonary diseases	Accuracy, 98%; Sensitivity, 87%; Specificity, 94%; Precision, 89%
Che Azemin et al. (58)	ResNet-101	5,982 chest x-ray images (154 COVID-19)	Identify COVID-19/non-COVID19	Accuracy, 71.9%; Sensitivity, 77.3%; Specificity, 71.8%; AUC, 0.82
Islam et al. (59)	CNN, LSTM	4,575 chest x-ray images (1,525 COVID-19)	Identify COVID-19/pneumonia/normal	Accuracy, 99.4%; Sensitivity, 99.3%; Specificity, 99.2%; AUC, 0.999; F1 score 98.9%
Jain et al. (60)	ResNet	1,215 chest x-ray images (250 COVID-19)	Identify COVID-19/viral pneumonia/bacterial pneumonia/normal	Accuracy, 97.77%; Sensitivity, 97.14%
Nour et al. (61)	CNN, Machine leaning	2,905 chest x-ray images (219 COVID-19)	Identify COVID-19/viral pneumonia/normal	Accuracy, 98.97%; Sensitivity, 89.39%; Specificity, 99.75%; F1 score 96.72%
Rahaman et al. (62)	CNN	860 chest x-ray images (260 COVID-19)	Identify COVID-19/pneumonia/normal	Accuracy, 89.3%; Sensitivity, 89%; Precision, 90%; F1 score 90%
Rahimzadeh et al. (63)	Xception, ResNet50V2	15,085 chest x-ray images (180 COVID-19)	Identify COVID-19/pneumonia/normal	Accuracy, 99.5%; Sensitivity, 80.53%
Rajaraman et al. (64)	CNN	14,997 chest x-ray images (286 COVID-19)	Identify COVID-19/viral pneumonia/bacterial pneumonia/normal	Accuracy, 99.01%; Sensitivity, 99.01%; AUC, 0.9972
Toraman et al. (65)	Convolutional CapsNet	2,331 chest x-ray images (231 COVID-19)	Identify COVID-19/normal	Accuracy, 97.24%; Sensitivity, 97.42%; Specificity, 97.04%; Precision, 97.06%; F1 score 97.24%
Ucar and Korkamz (66)	Deep Bayes-SqueezeNet	5,949 chest x-ray images (76 COVID-19)	Identify COVID-19/pneumonia/normal	Accuracy, 98.26%; Sensitivity, 98.26%; Specificity, 99.13%; F1 score 98.25%
Waheed et al. (67)	CovidGAN (ACGAN)	1,124 chest x-ray images (403 COVID-19)	Identify COVID-19/normal	Accuracy, 95%; Sensitivity, 90%; Specificity, 97%

*CNN, Convolutional Neural Network; LSTM, Long Short-Term Memory; ACGAN, Auxiliary Classifier Generative Adversarial Network*.

### Predicting the Prognosis of COVID-19

The ability to identify a patient's risk of deterioration during their hospitalization is critical for effective medical resource allocation and to ensure that patients receive appropriate management during the COVID-19 pandemic. We identified several AI models built on the chest CT images that accurately quantified lung abnormalities related to COVID-19 and evaluated the severity and prognosis of the disease (70, 76, 79, 80). Some studies showed that deep learning models could predict the risk of COVID-19 patients developing critical illness, based on clinical characteristics at hospital admission, such as clinical, laboratory and radiological characteristics (68, 71, 73–75, 77, 78). Iwendi et al. (69) developed a model using the geographical, traveling, health, and demographic data of COVID-19 patients to predict the severity and the possible outcomes of the cases. In general, AI models reached accuracy of 74.4 to 95.20%, sensitivity of 72.8 to 98.00%, specificity of 55 to 96.87% and AUC of 0.66 to 0.997 in predicting critical COVID-19 (Table 4). Accurately determining the prognosis of COVID-19 patients as early as possible and starting early treatment may improve their prognosis and reduce mortality from COVID-19.

**Table 4:** Application of AI in predicting prognosis of COVID-19.

**References**	**Algorithm**	**Subjects**	**Objective**	**Results**
Assaf et al. (68)	ANN, Random Forest and CRT	389 COVID-19 patients	Predict severity of COVID-19	Accuracy, 92%; Sensitivity, 88%; Specificity, 92.7%
Li et al. (70)	POI and iHU	196 COVID-19 patients	Predict severity of COVID-19	Sensitivity, 93.67%; Specificity, 88.05%; AUC, 0.97
Liang et al. (71)	Deep learning survival cox model	1,590 COVID-19 patients	Predict severity of COVID-19	Concordance index 0.894; AUC, 0.911
Yao et al. (75)	SVM	137 COVID-19 patients	Predict severity of COVID-19	Accuracy, 81.48%
Yu et al. (76)	DenseNet-201,SVM model	202 COVID-19 patients	Predict severity of COVID-19	Accuracy, 95.2%; Sensitivity, 91.87%; Specificity, 96.87%; AUC, 0.99
Iwendi et al. (69)	Random forest	–	Predict severity of COVID-19	Accuracy, 94%; Sensitivity, 75%; F1 score 86%
Abdulaal et al. (12)	ANN	398 COVID-19 patients	Predict mortality risk of COVID-19	Accuracy, 86.25%; Sensitivity, 87.50%; Specificity, 85.94%; AUC, 0.9012
Ma et al. (72)	Random Forest and XGboost	292 COVID-19 patients	Predict mortality risk of COVID-19	AUC 0.9521
Mushtaq et al. (73)	CNN	697 COVID-19 patients	Predict severity and mortality risk for COVID-19	For mortality, the AUCs were 0.66, for critical COVID-19, the AUCs were 0.77
Wu et al. (77)	LASSO logistic regression model	110 COVID-19 patients	Predict mortality risk of COVID-19	Sensitivity, 98%; Specificity, 91%; AUC, 0.997
Cheng et al. (78)	Random Forest	1,987 COVID-19 patients	Identify patients at risk of ICU transfer within 24 h	Accuracy, 76.2%; Sensitivity, 72.8%; Specificity, 76.3%; AUC, 0.799
Fu et al. (79)	LASSO, mRMR, SVM	64 COVID-19 patients	Identify the progression of COVID-19	Sensitivity, 80.95%; Specificity, 74.42%; AUC, 0.833
Wu et al. (74)	ADASYN, Logistic Regression	426 COVID-19 patients	Predict severity risk for COVID-19	Accuracy, 74.4–87.5%; Sensitivity, 75–96.9%; Specificity, 55–88%; AUC, 0.84–0.93
Xiao et al. (80)	MIL, ResNet34	408 COVID-19 patients	Predict severity risk for COVID-19	Accuracy, 81.9%; AUC, 0.892

*ANN, Artificial Neural Network; RF, Random Forest; CRT, Classification and Regression Decision Tree; POI, portion of infection; iHU, average infection Hounsfield unit; SVM, Support Vector Machine; LASSO, Least Absolute Shrinkage and Selection Operator; mRMR, Minimum Redundancy Maximum Correlation; ADASYN, Adaptive Synthetic Sampling; MIL, Multiple Instance Learning*.

### Predicting the Epidemic Trend of COVID-19

COVID-19 has spread globally and had a substantial impact. It was defined as a pandemic by the World Health Organization (WHO) in March 2020. As the COVID-19 pandemic evolves, it is vital to focus on building prediction models to help policymakers and health managers to allocate healthcare resources and prevent or limit outbreaks (82). We identified 9 studies that sought to predict the epidemic trend of COVID-19 (81–89) (Table 5). Of these, 6 studies used long short-term memory (LSTM) models with or without other models to predict the incidence, confirmed cases, deaths and recoveries, development trend and possible stopping time of COVID-19 (82, 84–86, 88, 89). Alsayed et al. (81) used the Susceptible–Exposed–Infectious–Recovered (SEIR) model combined with machine learning to predict the epidemic's evolution or estimate the unreported number of infections. Mollalo et al. (83) tested the applicability of multi-layer perceptron (MLP) artificial neural networks in simulating cumulative incidence of COVID-19 at the county-level across the continental USA. Shahid et al. (84) proposed prediction models including support vector regression (SVR), autoregressive integrated moving average (ARIMA), long short-term memory (LSTM) and Bi-directional long short-term memory (Bi-LSTM) to predict confirmed cases, deaths and recoveries in ten major countries affected by COVID-19. Zheng et al. (85) proposed an improved susceptible–infected (ISI) model to estimate the variety of the infection rates and to analyze the transmission laws and development trend. Ribeiro et al. (88) used several machine learning models to forecast the cumulative confirmed cases of COVID-19 in the ten Brazilian states with a high daily incidence, and rank the models based on their accuracy. The results of these studies may bring broad benefits by helping to control and prevent COVID-19.

**Table 5:** Application of AI in predicting the epidemic trend of COVID-19.

**References**	**Algorithm**	**Country**	**Objective**	**Results**
Alsayed et al. (81)	GA, SEIR, ANFIS	Malaysia	Estimate the infection rate, epidemic peak, and the number of infected cases	Infection rate is 0.228 ± 0.013, NRMSE 0.041, MAPE 2.45%, R2 of 0.9964
Ayyoubzadeh et al. (82)	LSTM, linear regression	Iran	Predict the incidence	RMSE: LSTM, 27.187 (SD 20.705); Linear regression, 7.562 (SD 6.492)
Mollalo et al. (83)	MLP neural network	The US	Predict incidence rates	RMSE, 0.722409; MAE. 0.355843; correlation coefficient 0.645481
Shahid et al. (84)	ARIMA, SVR, LSTM, Bi-LSTM	Ten countries	Predict confirmed cases, deaths, and recoveries	Bi-LSTM generates lowest MAE and RMSE values of 0.0070 and 0.0077 in China; r2_score 0.9997
Zheng et al. (85)	ISI, NLP, LSTM	China	Analyze the transmission laws and development trend	Obtain MAPEs with 0.52, 0.38, 0.05, and 0.86% for the next 6 days in Wuhan, Beijing, Shanghai, and countrywide, respectively
Arora et al. (86)	LSTM	India	Predict daily and weekly positive cases	Daily predictions MAPE <3% and weekly predictions MAPE <8%
Chimmula and Zhang (87)	LSTM	Canada	Predict the trends and possible stopping time of COVID-19	For short term predictions, RMSE, 4.83; accuracy, 93.4%. For long term predictions, RMSE, 45.70; accuracy, 92.67%
Ribeiro et al. (88)	SVR, stacking-ensemble learning, ARIMA, CUBIST, RIDGE, and RF	Brazil	Forecast the cumulative confirmed cases	sMAPE in a range of 0.87–3.51, 1.02–5.63, and 0.95–6.90% in 1, 3, and 6-days-ahead, respectively
Shastri et al. (89)	Variants of LSTM	India, The USA	Forecast the confirmed cases and death cases	Achieved accuracies of 97.82, 98, 96.66, and 97.50%, MAPE of 2.17, 2.00, 3.33, 2.50 for India confirmed cases, USA confirmed cases, India death cases and USA death cases, respectively

*GA, Genetic Algorithm; SEIR, Susceptible–Exposed–Infectious–Recovered; ANFIS, Adaptive Neuro-Fuzzy Inference System; LSTM, Linear regression and long short-term memory; R_0_, Reproductive number; MLP, Multilayer perceptron; RMSE, Root-mean-square error; NRMSE, Normalized root mean square error; MAE, Mean absolute error; ISI, Improved susceptible–infected; PSO, Particle Swarm Optimization; MAPE, Mean Absolute Percentage Error; NLP, Natural Language Processing; ARIMA, Autoregressive Integrated Moving Average; CUBIST, Cubist Regression; RF, Random Forest; RIDGE, Ridge Regression; SVR, Support Vector Regression*.

### Drug Discovery and Vaccine Development for COVID-19

With the spread of COVID-19 showing no signs of slowing and there are few proven effective therapeutics for COVID-19, thousands of people continue to die from the disease every day. It is essential to develop antiviral drugs and vaccines against SARS-CoV-2. It usually needs a long time to develop a drug or vaccine using traditional methods but to try to accelerate this process, several studies have applied AI techniques to identify potential drugs and develop effective and safe vaccines for COVID-19. We identified 9 studies that developed models to find potential drugs and vaccines for COVID-19(17, 90–97) (Table 6).

**Table 6:** Application of AI in drug discovery of COVID-19.

**References**	**Algorithm**	**Objective**	**Results**
Ke et al. (91)	DNN	Drug repurposing	Identified 80 marketed drugs with potential, there are 13 drugs with great potentials for further development toward treating COVID-19
Zeng et al. (92)	Deep-learning (CoV-KGE)	Drug repurposing	Identified 41 repurposable drugs (AUROC = 0.85)
Gao et al. (90)	GBDT model	Drug repurposing	Identified 20 drugs with potential (Pearson correlation coefficient, 0.78; RMSE, 0.792)
Stebbing et al. (93)	AI algorithms	Drug repurposing	Baricitinib can be used for COVID-19 infection
Zhang et al. (94)	DFCNN	Drug development	Provided potential compound and tripeptide lists for 2019-nCov_3Clike protease
Batra et al. (95)	Machine learning	Drug development	Identified 75 FDA-approved and 100 other ligands, molecular fragments and molecular descriptors
Joshi et al. (96)	RNN	Drug development	Found two compounds Palmatine and Sauchinone formed very stable complex with Mpro
Ton et al. (97)	Deep learning	Drug development	Screening 1.3 billion compounds from ZINC15 library to identify top 1,000 potential ligands for Mpro

*GBDT, Gradient-boosted decision trees; DFCNN, Dense Fully Convolutional Neural Network; RNN, Recurrent Neural Networks; Mpro, Main Protease*.

### Drug Repurposing

Drug repurposing refers to the application of approved drugs to new therapeutic indications, which has become a successful drug development strategy for reducing development costs and increasing the simplicity of drug approval procedures (99). AI algorithms could be trained and then be used to screen existing drugs that may prove effective in the treatment of COVID-19. Ke et al. (91) used AI to identify 13 drugs with activities against feline infectious peritonitis (FIP) coronavirus, and further studies proved their activities against SARS-CoV-2 in clinical applications. In another study, Zeng et al. (92) identified 41 high-confidence repurposed drug candidates with a higher area under the receiver operating characteristic (AUROC) of 0.85. Gao et al. (90) developed a gradient-boosted decision trees (GBDT) model for screening 8,565 drugs in DrugBank, finally finding 20 FDA-approved drugs and 20 investigational or off-market drugs that might be effective against SARS-CoV-2. Stebbing et al. (93) used AI prediction to identify Baricitinib, which is used to treat rheumatoid arthritis and myelofibrosis, can be used for COVID-19 infection through proposed anti-cytokine effects and as an inhibitor of host cell viral propagation.

### Drug Development

Zhang et al. (94) built a protein 3D model of 3CLpro and used a deep learning method to identify protein-ligand interacting pairs, and finally provided potential compound and tripeptide lists for 3CLpro. Batra et al. (95) combined machine learning and high-fidelity ensemble docking to identify 75 FDA-approved and 100 other ligands from drug data sets as potential therapeutic agents against COVID-19. Joshi et al. (96) used deep-learning models to screen natural compounds and found that two compounds Palmatine and Sauchinone formed very stable complex with Mpro, which may be considered for therapeutic development against the SARS-CoV-2. Ton et al. (97) developed Deep Docking (DD) to screen 1.3 billion compounds from ZINC15 library and identify top 1,000 potential ligands for SARS-CoV-2 Mpro protein.

### Vaccine Development

Without an existing effective medical therapy, the development of an effective and safe vaccine is an important method to deal with this highly infectious disease caused by the SARS-CoV-2 coronavirus. Ong et al. applied a ML tool to predict the S protein, nsp3, 3CL-pro, and nsp8-10 were crucial to the viral adhering and host invasion by investigating the entire proteome of SARS-CoV-2. SARS-CoV-2 S protein has the highest protective antigenicity score and was identified as the most favorable vaccine candidate, besides, the nsp3 protein was selected for further investigation (17). The predicted vaccine targets have the potential for COVID-19 vaccine developed, however, they need to be further evaluated in clinical studies.

## Discussion

Our systematic review includes 78 articles on the application of AI for COVID-19. These spanned radiological diagnosis, prediction of prognosis, estimation of epidemic trends and drugs and vaccines discovery for COVID-19.

The gold standard for diagnostic tests for COVID-19 is real-time reverse-transcriptase polymerase chain reaction (RT-PCR). However, RT-PCR does produce false negatives or fluctuating results (100). A study compared the diagnostic performance of chest computed tomography (CT) scan with RT-PCR and found that the chest CT is more sensitive than RT-PCR (98 vs. 71%, respectively, *P* < 0·001) (101), suggesting that Chest CT could be a supplementary diagnostic measure to help physicians make faster and more accurate decisions. AI technique is used for identifying or classifying images, recognizing speech and processing natural language (102). It is well-suited to developing tools to assist with the use of chest CT to diagnose COVID-19 (103). Advanced AI-based algorithms can learn the typical CT image signs, such as bilateral and subpleural ground-glass opacities (GGO), vascular thickening, spider web, and even crazy-paving patterns (104). In addition, the algorithms can also learn some high-dimensional features that radiologists cannot handle, such as texture and wavelet information, thereby allowing pneumonia caused by SARS-CoV-2 to be distinguished from that caused by other pathogens, through advanced AI-based algorithms (36). Several studies have shown that deep learning can automatically differentiate COVID-19 from non-COVID-19 or other pneumonia diseases through extracting features from chest CT and X-rays images. As shown in Tables 2, 3, most of the studies achieved over 90% accuracy, sensitivity and specificity. The performance of AI-assisted diagnosis was comparable to radiologists with significant clinical experience and could assist and improve the performance of radiologists. This means that AI-assisted diagnosis is a useful screening tool, which might shorten patient waiting time, simplify the workflow, reduce the workload of radiologists and allow them to respond more quickly and effectively in emergencies (38). AI techniques have recently shown great potential in the real-time diagnosis of COVID-19 by using images. However, the severity of disease, comorbidities, and the proportion of asymptomatic patients have an impact on the diagnostic sensitivity of chest CT (105). Chest CT have a relatively high sensitivity in symptomatic COVID-19 patients, but low specificity (106). The Italian Society of Medicine and Interventional Radiology recommends that CT should be used as a screening tool only for symptomatic patients with specific indications (107). AI should be used to assist diagnosis, not an independent diagnostic tool. Second, the evaluation of patients based on a single data type may be biased, therefore, AI-assisted diagnosis needs to be used in combination with other laboratory tests and a multimodal AI framework was required to analyze different data types (108). Third, several studies used a relatively small amount of data to train the deep learning models, and the testing data set had the same sources as the training data set. This may cause the problem of overfitting of the models (108, 109). Fourth, there is little evidence to directly compare the performance of humans and machines or the performance of AI in actual clinical work. Only Bai, Song, Ni, and a latest research show that AI assistance improved radiologists' performance in identifying COVID-19(13, 14, 41, 42). In addition, confounding factors can influence the internal validity of researches and the accuracy of AI-based radiological interpretation, such as the variation of respiratory effort, image contrast, technique, and resolution of radiological images (110).

In regard to the prognosis of patients with COVID-19, information available at hospital admission, typically 6 days (median) before the patient developed severe COVID-19, can be used by AI for early detection of patients at higher risk, allowing adjustments to their in-hospital allocation and management (68). AI can evaluate the prognosis of COVID-19 patients by clinical manifestations, laboratory and radiological characteristics and identify potential predictive biomarkers related to the disease's severity. The significantly elevated LDH levels reflects the severity of pneumonia, and increased serum CRP predicts the risk of death in patients with severe COVID-19 (72). Age and comorbidities may be risk factors for severe COVID-19 after hospitalization, such as diabetes, hypertension and cardiovascular diseases (74). As well as chest CT images being a powerful tool to assist clinical diagnosis because of its high sensitivity and the ability to quantify the COVID-19 associated lung abnormalities, they also help assess the severity and prognosis of the disease and monitor the development of the disease (70, 76, 80). Accurate risk prediction of patients with critical COVID-19 may help to optimize patient triage and in-hospital allocation, monitor disease progression and treatment response, prioritize medical resources and improve the overall management of the COVID-19 pandemic (68). However, several of the studies we identified were retrospective single-center studies, which reduces their external validity. Therefore, the results in this review may not be generalizable to other environment and healthcare systems, especially considering the high variability of COVID-19 in different countries and populations (68, 72). In addition, prospective studies with a larger number of patients from multiple locations are required to verify the predictive ability of a model (81).

Many statistical and numerical models have been used to predict the trend of the COVID-19 pandemic, such as the epidemic peak, transmission and development trend, the SEIR model is one of the most popular models (81). Alsayed et al. combined the SEIR model with machine learning to characterize the epidemic dynamics and to predict possible contagion scenarios of COVID-19 in Malaysia (81). Long short-term memory (LSTM) is a recurrent neural network that is an effective model for the prediction of time series where data are sequential (82). Therefore, LSTM has been widely used to predict the confirmed cases, death and recovery, development trend of COVID-19 through time (82, 84–87, 89). Embed the NLP and LSTM into the Improved susceptible–infected (ISI) model is more accurate and reliable than the traditional epidemic model, providing a basis for estimating the law of virus transmission (85). In addition, AI models, such as SVR, stacking-ensemble learning, ARIMA, CUBIST, RIDGE, RF and MLP also play an important role in estimating the epidemic of COVID-19 (83, 88). AI techniques provide useful tools to help policy makers make decisions and take actions to prevent diffusion at the early stage of the epidemic and to minimize the subsequent impact of COVID-19. However, we cannot verify and validate the database, so it is difficult to compare and calibrate results with other studies.

Traditional drug repurposing design methods are based on repeated trials and there is no systematic way to screen the enormous drug-dose parameter space (111). AI is an effective approach to quickly detect potential drugs as antiviral therapeutics for COVID-19 (91). Deep learning, using the relationship between drug targets and diseases can be used as a helpful tool to assist drug repurposing and minimize the possibility of failure in clinical trials (92). Chymotrypsin-like protease is a major therapeutic target, and several studies used it to identify potential therapeutic drugs against COVID-19 by performing drug screening over protein-ligand or protein-peptide among existing drugs (90, 94, 112). In addition, using AI techniques to conduct virtual screening of biologically active compounds can support new drug discovery. However, all predicted drugs must be tested in randomized trials before being used in COVID-19 patients (92).

During the COVID-19 pandemic, an effective and safe vaccine is essential to prevent infection and reduce deaths. The development of vaccines was a complicated process with many difficulties, such as the complexity of the human immune system and the variability between different populations (113). Research organizations in many nations and multinational companies are developing various vaccines, including whole virus vaccine, subunit vaccine, nucleic acid vaccines. Researchers are trying to use AI techniques to explore the vaccine development. Ong et al. (17) predict 6 vaccine candidates, including S protein, nsp3, 3CL-pro, and nsp8-10, S protein was identified as the most favorable vaccine candidate. Nsp3 has a high antigen protection score and has not been used for vaccine development, therefore it was also selected for further investigation. Currently, S protein has been widely used in subunit vaccines, and other proteins are expected to be used in vaccine development. In addition, the latest research analyzed the entire SARS-CoV-2 proteome via AI and identified some of the epitope hotspots that can be used in vaccine formulations (114).

AI has the potential to be an important tool in the fight against COVID-19 and similar pandemics. However, there were many problems in using AI to predict and diagnose COVID-19, and rigorous clinical trials were required before drugs and vaccines developed by AI are approved, so the use of AI has so far been rather limited. It requires continuous efforts by researchers. But recent studies have shown that AI tools such as computer vision and robots have the potential to be widely promoted and used in the short term, such as infrared thermal cameras have been paired with AI-powered facial recognition systems to determine if the individual are wearing masks, using camera images to observe whether social distancing rules are complied, AI-based dialogue chatbots can complete symptom screening and patient education (19). Robotics, AI, and digital technology have been implemented in sanitation for hospitals and public areas, delivery in hospitals and public spaces, patrolling, screening, health consulting, and virus tracking (115).

Our systematic review has several limitations that should be noted. First, although we conducted a systematic search, we only included articles published in English, introducing the possibility of publication bias. Second, in the included studies, the models in 60 studies were at high risk of bias according to assessment with PROBAST (Table 1), and the remaining 18 studies were not evaluated due to lack of suitable evaluation tools. Therefore, the predictive performance of these AI models when used in practice is probably lower than that reported, which means that the predictions of these models may be unreliable. Third, many studies had small sample sizes, and the testing data set had the same sources as the training data set, which leads to an increased risk of overfitting. Fourth, over one fifth of the studies were retrospective single-center studies, which might limit their applicability to the specific center or the same geographical region. This means that results may not be generalizable to other settings and places.

## Conclusions

Artificial intelligence has been widely explored in the medical field, especially for enhancing medical and healthcare capabilities. At present, many countries continue to struggle to contain the spread of COVID-19. Facing limited medical resource and increasing healthcare pressure, the use of AI techniques to assist with diagnosis, treatment, prediction of prognosis, evaluation of epidemic trends, surveillance and public health decision-making may improve the efficiency and ability of humans to fight the COVID-19 pandemic.

## Data Availability Statement

The original contributions presented in the study are included in the article/Supplementary Material, further inquiries can be directed to the corresponding author/s.

## Author Contributions

YZ and LW conceived of the study. LW and DW screened the literature for relevancy and did the data extraction. SZ, JH, and LZ did the quality appraisal. XT and TL resolved any disagreements in study relevancy, extraction, and quality appraisal. LW and LC drafted and revised the manuscript. HF, YZ, and MC directed and revised the manuscript. All authors participated in data interpretation and revised the manuscript for intellectual content.

## Funding

This study was supported by National Key R&D Program of China (2017YFC1309703), 1·3·5 project for disciplines of excellence–Clinical Research Incubation Project, West China Hospital, Sichuan University (2019HXFH008), and Science & Technology Department of Sichuan Province (2020YFS0186). The funders of this research did not contribute to the study design, data analysis, data interpretation or preparation of the manuscript.

## Conflict of Interest

The authors declare that the research was conducted in the absence of any commercial or financial relationships that could be construed as a potential conflict of interest.

## Publisher's Note

All claims expressed in this article are solely those of the authors and do not necessarily represent those of their affiliated organizations, or those of the publisher, the editors and the reviewers. Any product that may be evaluated in this article, or claim that may be made by its manufacturer, is not guaranteed or endorsed by the publisher.
