# Supplementary material for: Artificial Intelligence for COVID-19: A Systematic Review
Source: Front Med (Lausanne). 2021 Sep 30;8:704256. doi: 10.3389/fmed.2021.704256 (PMC8514781; doi:10.3389/fmed.2021.704256)
Supplement: Supplementary file 1 [file Data_Sheet_1.DOCX]

Supplementary Material 1 The details of the PubMed search strategy for the systematic review

(((Artificial Intelligence[MeSH Terms]) OR ((((((((((((((((Intelligence, Artificial[Title/Abstract]) OR (Computational Intelligence[Title/Abstract])) OR (Intelligence, Computational[Title/Abstract])) OR (Machine Learning[Title/Abstract])) OR (Deep Learning[Title/Abstract])) OR (Predictive Model[Title/Abstract])) OR (Machine Intelligence[Title/Abstract])) OR (Intelligence, Machine[Title/Abstract])) OR (Computer Reasoning[Title/Abstract])) OR (Reasoning, Computer[Title/Abstract])) OR (Computer Vision Systems[Title/Abstract])) OR (Computer Vision System[Title/Abstract])) OR (System, Computer Vision[Title/Abstract])) OR (Systems, Computer Vision[Title/Abstract])) OR (Vision System, Computer[Title/Abstract])) OR (Vision Systems, Computer[Title/Abstract]))) AND ((("COVID-19" [Supplementary Concept]) OR (((((((((((2019 novel coronavirus disease[Title/Abstract]) OR (COVID19[Title/Abstract])) OR (COVID-19 pandemic[Title/Abstract])) OR (SARS-CoV-2 infection[Title/Abstract])) OR (COVID-19 virus disease[Title/Abstract])) OR (2019 novel coronavirus infection[Title/Abstract])) OR (2019-nCoV infection[Title/Abstract])) OR (coronavirus disease 2019[Title/Abstract])) OR (coronavirus disease-19[Title/Abstract])) OR (2019-nCoV disease[Title/Abstract])) OR (COVID-19 virus infection[Title/Abstract])))))
