# Supplementary material for: Artificial Intelligence for COVID-19: A Systematic Review
Source: Front Med (Lausanne). 2021 Sep 30;8:704256. doi: 10.3389/fmed.2021.704256 (PMC8514781; doi:10.3389/fmed.2021.704256)
Supplement: Supplementary file 3 [file Data_Sheet_3.DOCX]

Supplementary material 3 PROBAST (prediction model risk of bias assessment tool) statement

# Participants

- 1. Were appropriate data sources used, e.g., cohort, RCT, or nested case–control study data?
  2. Were all inclusions and exclusions of participants appropriate?

# Predictors

- 1. Were predictors defined and assessed in a similar way for all participants?
  2. Were predictor assessments made without knowledge of outcome data?
  3. Are all predictors available at the time the model is intended to be used?

# Outcome

- 1. Was the outcome determined appropriately?
  2. Was a prespecified or standard outcome definition used?
  3. Were predictors excluded from the outcome definition?
  4. Was the outcome defined and determined in a similar way for all participants?
  5. Was the outcome determined without knowledge of predictor information?
  6. Was the time interval between predictor assessment and outcome determination appropriate?

# Analysis

- 1. Were there a reasonable number of participants with the outcome?
  2. Were continuous and categorical predictors handled appropriately?
  3. Were all enrolled participants included in the analysis?
  4. Were participants with missing data handled appropriately?
  5. Was selection of predictors based on univariable analysis avoided?†
  6. Were complexities in the data (e.g., censoring, competing risks, sampling of control participants) accounted for appropriately?
  7. Were relevant model performance measures evaluated appropriately?
  8. Were model overfitting, underfitting, and optimism in model performance accounted for?†
  9. Do predictors and their assigned weights in the final model correspond to the results from the reported multivariable analysis?†

RCT = randomized controlled trial;

† Development studies only.
